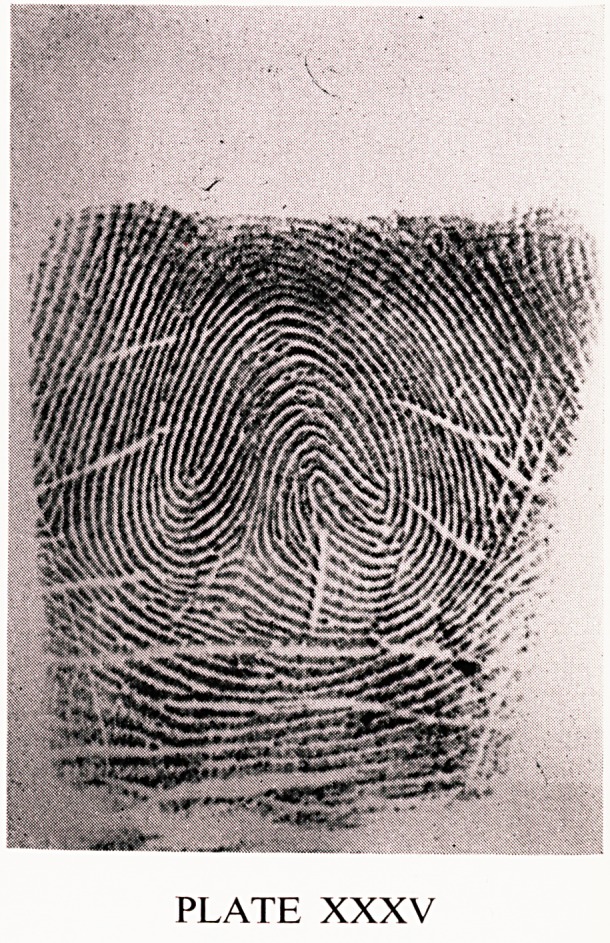# Finger-Printing in Congenital Heart Disease

**Published:** 1969-10

**Authors:** T. J. David


### FINGERPRINTS IN CONGENITAL HEART DISEASE

**T. J. David**

There are three situations in which congenital heart disease (CHD) may occur:

Firstly, there are syndromes with abnormal chromosomes in which CHD is common, but is only one of the abnormalities. An example is Down's anomaly, in which 40 to 56% of cases have CHD, usually an A-V canal or a ventricular septal defect. Secondly, there are hereditary disorders with normal chormosomes in which CHD is common. An example is Marfan's syndrome, which is often associated with aortic or mitral regurgitation. Thirdly, there is isolated CHD, which may be idiopathic or inherited. Conditions that may be inherited as autosomal dominants include supravalvular aortic stenosis without hypercalcaemia, secundum atrial septal defect and cardiomyopathy. About 1 in 20 cases of CHD are associated with CHD in a parent or sibling. When CHD is present in both of a pair of twins, the lesions are almost always different.

Fingerprints are known to have a genetic basis. It has been suggested that finger and palm prints might be of practical use in CHD, firstly for genetic counselling, and secondly for diagnosis. There is considerable evidence that fingerprints might enable one to detect the familial cases of CHD, with which there is a considerable risk of further siblings being affected. There is no published evidence to suggest that fingerprints can help with diagnosis.

So far in the Bristol study, over 150 children and adults with CHD have had their finger and palm prints taken, and sometimes their sole and toe prints also. As many relatives as possible were also printed. Detailed family histories were recorded. The approach, in simple terms, was to see if the familial cases of CHD in each diagnostic group had any patterns in common. The fingerprints were taken and analysed in the same way as the police do it.

The human palms and soles are covered with ridged skin. These ridges are seen to run parallel with one another in small areas. Where three such areas meet, a triradius is said to be formed (Plate XXVIII). Triradii are the most useful landmarks for the classification of patterns on the fingers, and on the palms for the diagnosis of some chromosomal abnormalities.

Another pattern is an arch (Plate XXIX). As the name implies, the ridges run straight across the pattern in an arch-like fashion. There is no triradius. A third is a loop (Plate XXX). The ridges re-curve through 180 degrees and thus form a loop pattern. There is one triradius. There are two types of loops. In one type, the loop opens out onto the ulnar border of the finger and is therefore an ulnar loop. In the other the loop opens out onto the radial border of the finger and is a radial loop. Loops are further subclassified into groups A to K. A fourth is a whorl (Plate XXXI). The ridges run in a circular pattern and there are two triradii. Plate XXXII shows a twinned loop. Twinned loops have two well defined loops which more or less embrace one another. There are two triradii. One loop is the ascending loop, and the other is the descending loop. The two triradii are always on either side of the ascending loop. This classification is a simplified one. In fact the fingerprints were classified into 13 different primary patterns.

The following are the results for one diagnostic group, pulmonary valvular stenosis. There were 8 cases. In 2 of these cases first degree relatives were affected with different congenital heart lesions. The distribution of patterns in the 6 non-familial cases was normal. However, in the familial cases there were 12 arches (8 in one and 4 in the other). This is 36 times as many arches as in the other cases. The chances of 2 people having 12 arches is 1 in 119 in our control group of nurses, doctors, and medical students. This is statistically highly significant.

The conclusion that can be drawn from these figures is that familial CHD shows a great preponderance of arches. It suggests that further cases of pulmonary stenosis with, say, 4 or more arches, are likely to be familial. There is no evidence to suggest that fingerprints can be of use to diagnose pulmonary stenosis from these or any other figures.

As far as the other diagnostic groups are concerned, most of them are not large enough, or there are not enough genetic cases, to give any clear indication as to whether each diagnostic group is associated with a familial fingerprint pattern of its own. It seems likely that ventricular septal defects will produce a familial fingerprint pattern, and perhaps also Fallot's tetralogy. As far as can be seen from several familial atrial septal defects, they do not have any patterns in common.

It was never planned to investigate the possibilities of using fingerprints for the diagnosis of CHD, as it did not appear to be possible. However, after about a year of the survey, it was noticed that our collection had a great excess of three very rare fingerprint patterns.

As explained before, loops are sub-classified into groups A to K. Groups A to J are all common. The so-called K loop is so rare that in a detailed analysis of fingerprints of 1000 criminals by Scotland Yard, K loops are not quoted. Plate XXXIII shows such a K loop. The ridges bend over rather like a drooping flower, and these patterns are sometimes called nutant loops. They are commonest on the index fingers and thumbs. They are almost always radial. They are similar to the next pattern. Plate XXXIV shows a lateral pocket loop. As in the twinned loop there are two loops, an ascending and a descending one. The difference is that in a lateral pocket loop the triradii are on the same side of the ascending loop, where as in the twinned loop the triradii are on different sides. Lateral pockets are found in 1.5% of criminals, and in 0.18% of policemen. Plate XXXV shows a composite. There are many different types of composites. This one has three triardii, and also three loops. Composites are slightly more common than lateral pockets. They are both usually found on the index finger, or occasionally on the middle finger.

As far as the first 150 cases of CHD are concerned, there were 6 of these rare patterns amongst them, which is 2.5%. The interesting fact was that all these rare patterns were in one single diagnostic group, 5 cases of multiple cardiac abnormalities. 1 patient had two such rare patterns. Not one was present in the simple types of CHD. This would suggest that to find such a rare pattern in a case of CHD would suggest that there are multiple abnormalities. This has obvious practical applications. In one case, a little girl was operated on for a catheter-proven uncomplicated ventricular septal defect. She died shortly after operation, and at autopsy was found to have a bizarre abnormality of her mitral valve. She also had a lateral pocket loop.

The next finding was that there were 15 of these rare patterns in the

PLATE XXVIII

PLATE XXIX

PLATE XXX

PLATE XXXI

PLATE XXXII

PLATE XXXIII

PLATE XXXIV

PLATE XXXV

relatives of patients with CHD of a simple variety. It would have seemed reasonable to explain the rare patterns in the multiple-abnormality group as being abnormal fingerprints which went wrong at the same time as the heart. This cannot be true since, as has been found, they are also present in the normal relatives of simple cases of CHD. This paradox is difficult to explain as it does not follow any simple genetic rules. It is complicated by the fact that one such composite pattern has been found in a 16 year old girl with a hare lip and carcinoma of the colon with multiple metastases. She did not have polyposis coli. Also, 4 such patterns have been found in one person with a normal heart but unusual facies.

In conclusion, the results of a preliminary study of fingerprints and allied phenomena in CHD would suggest the following:—

1. Fingerprints may be a useful clue to indicate those with multiple cardiac abnormalities.

2. It may be possible to detect a familial association in certain types of CHD by examining the fingerprints. This would be particularly useful in cardiomyopathy. An analysis of palm prints suggests that they may contribute to the detection of the familial cases.

Insufficient numbers of most diagnostic groups have been obtained so far to draw any certain conclusions. Patients for cardiac catheterisation and open heart surgery will continue to be printed.

## Figures and Tables

**PLATE XXVIII f1:**
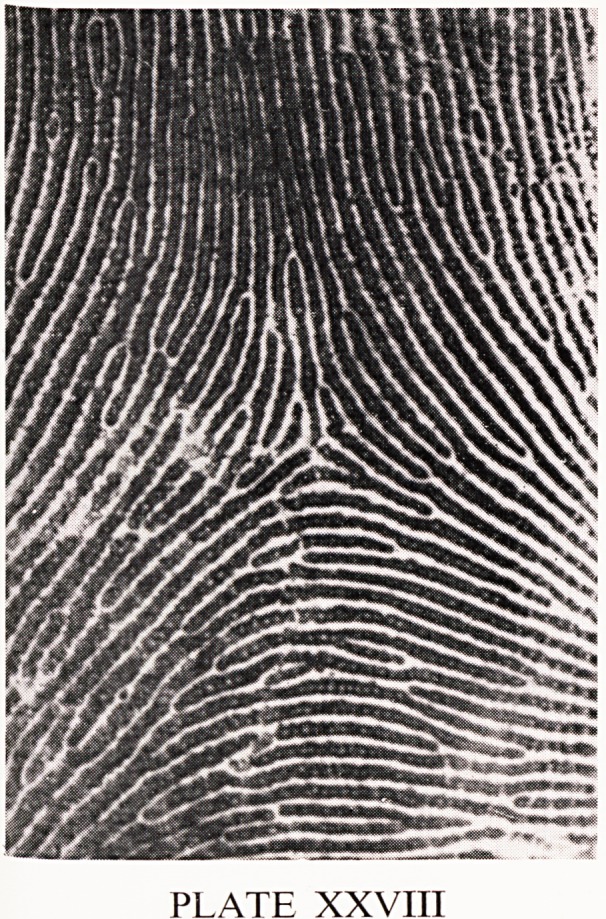


**PLATE XXIX f2:**
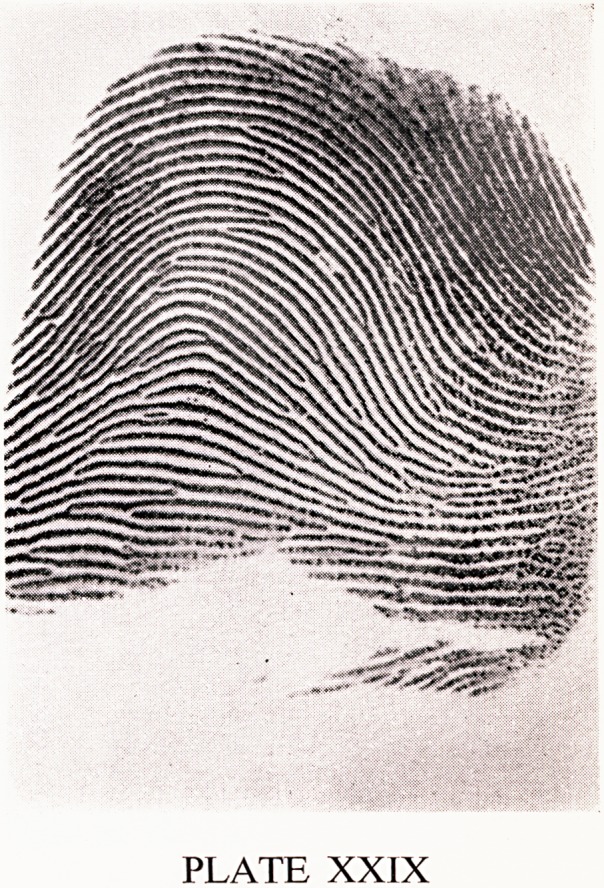


**PLATE XXX f3:**
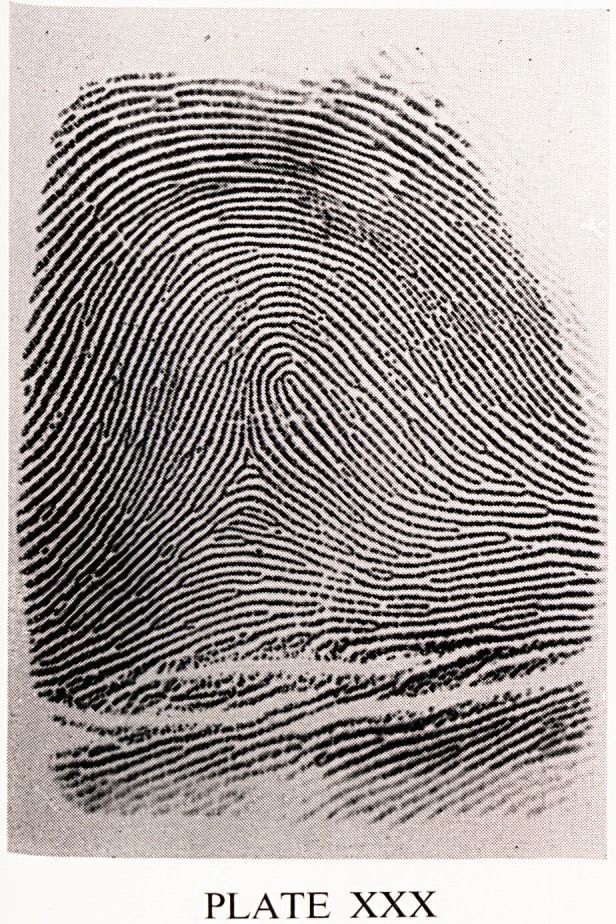


**PLATE XXXI f4:**
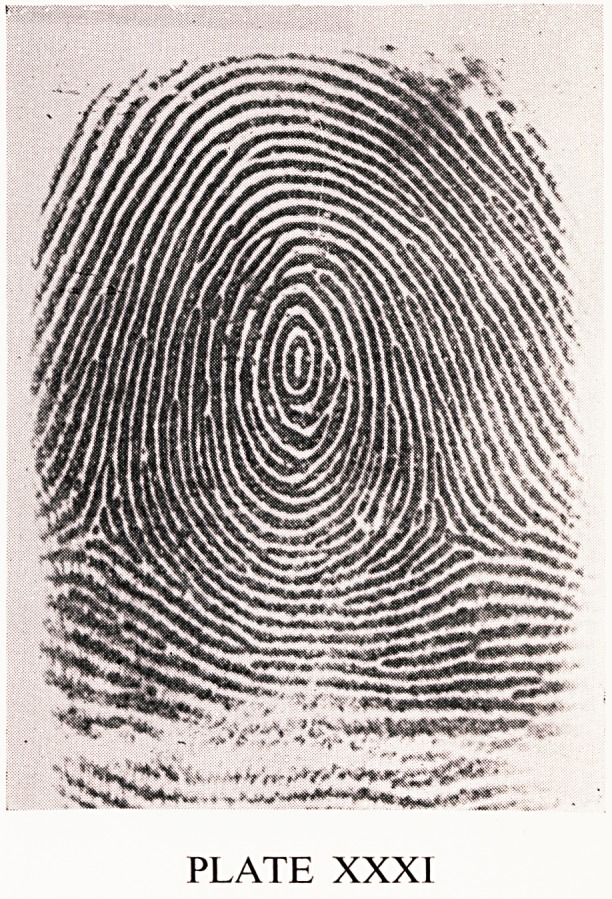


**PLATE XXXII f5:**
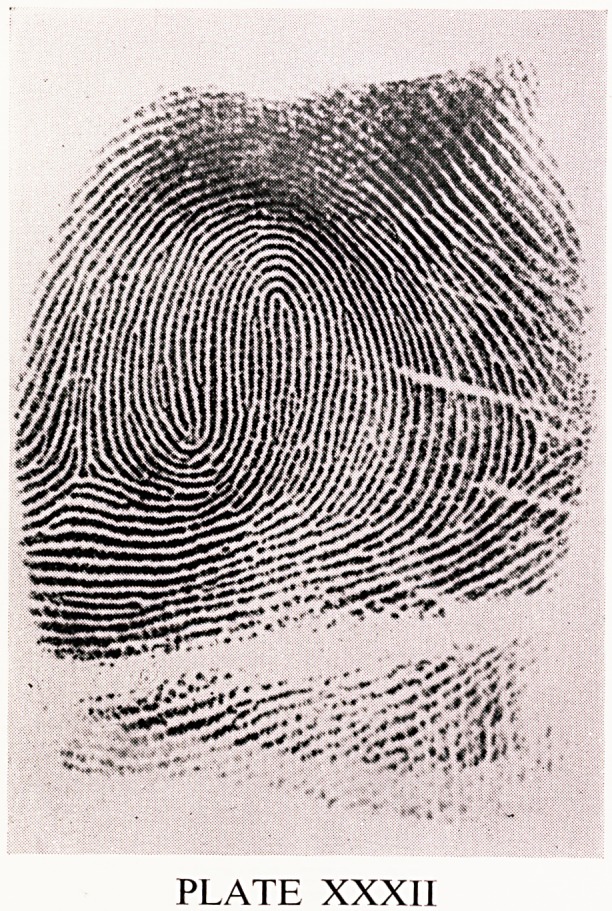


**PLATE XXXIII f6:**
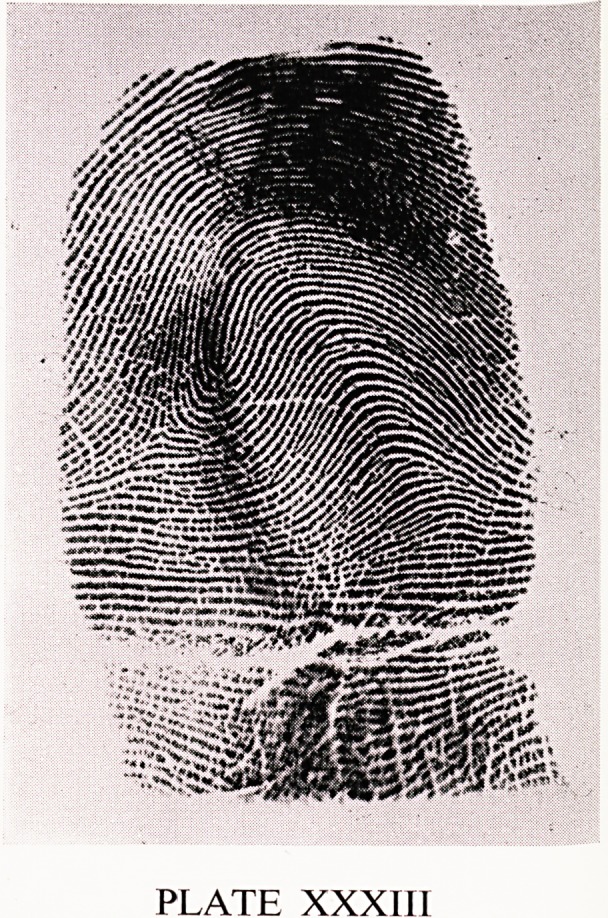


**PLATE XXXIV f7:**
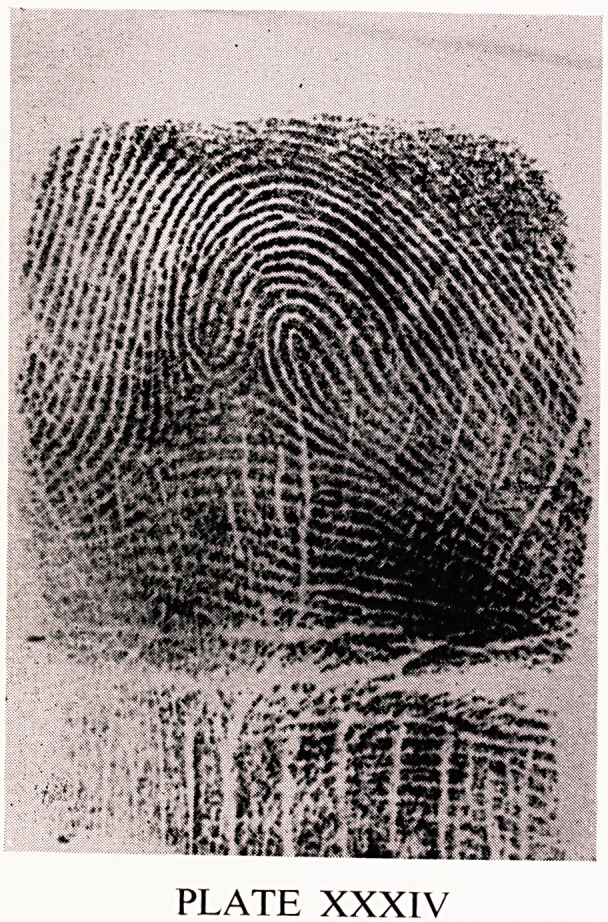


**PLATE XXXV f8:**